# Developmental Dioxin Exposure Alters the Methylome of Adult Male Zebrafish Gonads

**DOI:** 10.3389/fgene.2018.00719

**Published:** 2019-01-11

**Authors:** Camille Akemann, Danielle N. Meyer, Katherine Gurdziel, Tracie R. Baker

**Affiliations:** ^1^Department of Pharmacology, Wayne State University, Detroit, MI, United States; ^2^Institute of Environmental Health Sciences, Wayne State University, Detroit, MI, United States; ^3^Applied Genome Technology Center, School of Medicine, Wayne State University, Detroit, MI, United States

**Keywords:** 2, 3, 7, 8-tetrachlorodibenzo-*p*-dioxin, DNA methylation, zebrafish, epigenetics, reproduction, endocrine disrupting compounds

## Abstract

2,3,7,8-Tetrachlorodibenzo-*p*-dioxin (TCDD) is a persistent environmental toxicant and endocrine disrupting compound with reproductive and developmental effects in humans and model organisms, including zebrafish. Our previous microarray and histological studies found defects in spermatogenesis and fertility of zebrafish in response to acute developmental TCDD exposure. These effects are apparent following exposure during reproductive development, modeling fetal basis of adult-onset disease. Some outcomes of these previous studies (reduced fertility, changes in sex ratio, transcriptomic alterations) are also transgenerational – persisting to unexposed generations – through the male germline. We hypothesized that DNA methylation could be a possible mechanism for these reproductive effects and performed whole genome bisulfite sequencing (WGBS), which identifies whole genome DNA methylation status at the base pair level, on testes of adult zebrafish exposed to TCDD (two separate hour-long exposures to 50 pg/mL TCDD at 3 and 7 weeks post fertilization). In response to TCDD exposure, multiple genes were differentially methylated; many of which are involved in reproductive processes or epigenetic modifications, suggesting a role of DNA methylation in later-life health outcomes. Additionally, several differentially methylated genes corresponded with gene expression changes identified in TCDD-exposed zebrafish testes, indicating a potential link between DNA methylation and gene expression. Ingenuity pathway analysis of WGBS and microarray data revealed genes involved in reproductive processes and development, RNA regulation, the cell cycle, and cellular morphology and development. We conclude that site-specific changes in DNA methylation of adult zebrafish testes occur in response to acute developmental TCDD exposure.

## Introduction

2,3,7,8-Tetrachlorodibenzo-*p-*dioxin (TCDD), a by-product of industrial processes such as fossil fuel combustion, waste incineration, and metal production, is a widespread and persistent environmental toxicant. As a well-characterized model aryl-hydrocarbon receptor agonist, this endocrine disrupting compound affects a variety of organ systems, leading to human health outcomes including cancer, type II diabetes, and metabolic disease (Steenland et al., 1999; Manikkam et al., 2012; Warner et al., 2013). People exposed to TCDD suffer decreased semen quality, have longer menstrual cycles, and produce more female than male offspring (i.e., altered sex ratios) (Signorini et al., 2000; Eskenazi et al., 2002; Mocarelli et al., 2008). Similar reproductive outcomes have been demonstrated in model organisms including zebrafish (King Heiden et al., 2009; Manikkam et al., 2012; Baker et al., 2013).

Due to their short generation time, high fecundity, and externally developing embryos, zebrafish are an excellent transgenerational model for studying the reproductive and developmental outcomes of chemical exposure. Our earlier research demonstrated impaired fertility in adult zebrafish in response to TCDD exposure during sexual differentiation (two separate hour-long exposures to 50 pg/mL TCDD at 3 and 7 weeks post fertilization), modeling fetal basis of adult-onset disease (Baker et al., 2013). Several reproductive outcomes (altered sex ratio, decreased egg release, and transcriptional changes in reproductive genes) persisted through two subsequent generations (Baker et al., 2014a,b, 2016; Meyer et al., 2018). Reduced reproductive capacity was characterized by decreases in both egg release (a product of courtship between male and female zebrafish) and percentage of fertilized eggs, which presented across three generations (F_0_–F_2_). We determined these effects were male-mediated by outcrossing control and exposed lineage fish from each generation; only males from the exposed lineage demonstrated transgenerational reproductive effects (Baker et al., 2014b). Subsequent microarray and histologic analyses revealed differentially expressed genes involved in reproduction and defects in spermatogenesis, indicating a possible mechanism for the male-mediated decreased fertility (Baker et al., 2016; Meyer et al., 2018).

Parental exposures to environmental chemicals and other stressors, including paternal exposures, can contribute to adverse outcomes in offspring and subsequent generations (Jirtle and Skinner, 2007; Rodgers et al., 2013; Robledo et al., 2015). The mechanisms of adult-onset and transgenerational TCDD effects are unknown but thought to be epigenetic as studies have demonstrated changes in common epigenetic mechanisms such as DNA and histone methylation, as well as differential expression of DNA methyltransferases in response to TCDD exposure (Manikkam et al., 2012; Olsvik et al., 2014; Aluru et al., 2015; Ma et al., 2015; Baker et al., 2016; Sanabria et al., 2016). Because the DNA methylation pattern in zebrafish is passed down paternally through the sperm (Potok et al., 2013; Jiang et al., 2014), inheritance of epimutations in the DNA methylome is a promising mechanism of transgenerational male-mediated reproductive defects resulting from TCDD exposure. Other studies examining DNA methylation in zebrafish in response to TCDD exposure (Olsvik et al., 2014; Aluru et al., 2015) focused on either locus-specific or global assays, both of which have limitations for informing DNA methylation status. Reduced representative bisulfite sequencing improves on these methods, but still only focuses on a subset of CpG islands and promoter regions. In this study, we utilize whole genome bisulfite sequencing (WGBS) which provides base pair level DNA methylation status of the entire genome, eliminating potential masking of bidirectional changes in global analysis and providing more genome wide coverage than locus specific methods (Doherty and Couldrey, 2014).

The purpose of this study is to utilize WGBS to expand the current knowledge of TCDD-induced epigenetic effects, demonstrating that sublethal TCDD exposure during zebrafish gonadal differentiation and maturation causes changes in DNA methylation at specific genome loci which persist to adulthood. As our previous work revealed transcriptomic alterations in zebrafish testes induced by TCDD exposure, we also interpret our findings of changes in the testicular methylome within the context of this data (Baker et al., 2016). To our knowledge, we have performed the first WGBS on zebrafish in response to TCDD exposure. These data will provide valuable insight into the epigenetic effects of environmental endocrine-disrupting compounds as potential mechanisms of adult-onset and multigenerational disease.

## Materials and Methods

### Animal Husbandry

Zebrafish (AB strain) were kept at 28°C in buffered reverse osmosis water (60 mg/L Instant Ocean Salts; Aquarium Systems, Mentor, OH, United States) with a standard light/dark cycle of 14/10 h and fed Aquatox Fish Diet flakes (Zeigler, PA, United States) twice per day, supplemented with brine shrimp. Fish were raised in beakers with daily water changes of 40–60% at a density of five fish per 400 mL beaker between 3 and 6 weeks post-fertilization (wpf), and five fish per 800 mL beaker between 6 and 9 wpf. Adult fish were raised on a recirculating system at a maximum density of five fish per liter until euthanization at 1 year post fertilization. Fish were euthanized with tricaine methanesulfonate (1.67 mg/mL). Animal use protocols were approved by the Institutional Animal Care and Use Committees at Wayne State University and the University of Wisconsin-Madison, according to the National Institutes of Health Guide to the Care and Use of Laboratory Animals (Protocol No. M00489).

### TCDD Exposure

Exposures were performed as previously described in Baker et al. (2013). TCDD (>99% purity; Chemsyn) was used as a 0.4 ng/μL stock solution in dimethyl sulfoxide (DMSO). Zebrafish were exposed at 3 wpf and again at 7 wpf to waterborne TCDD (50 pg/mL) or vehicle (0.1% DMSO) for 1 h in glass beakers with gentle rocking. The number of fish per volume of dosing solution was 1 fish/mL at 3 wpf and 1 fish/2 mL at 7 wpf. All results are derived from three independent TCDD exposure experiments performed in successive blocks. The fish in the current experiment were collected and stored from the same blocks of the previously published microarray and histology studies (Baker et al., 2013, 2016).

### DNA Isolation

Testes were extracted from 1-year-old TCDD- and DMSO vehicle control-treated zebrafish and flash frozen in liquid nitrogen. Samples were kept at -80°C until DNA isolation. DNA isolation was performed on four DMSO control and four TCDD exposed samples using the BioRobot EZ1 workstation following the provided protocol from Qiagen (Hilden, Germany). Concentration and quality of DNA was measured using Qubit (Invitrogen, Carlsbad, CA, United States) and DropSense 96 (Trinean, Gentbrugge, Belgium), respectively (Supplementary Table S1). DNA was collected from different fish than those used for RNA collection and microarray analysis.

### Microarray

Microarray data were previously analyzed according to Baker et al. (2016). Microarray was performed on F_0_ zebrafish testes. Data were uploaded to NCBI GEO database (GSE77335). Microarray data were analyzed with one-way between subject ANOVA using the Transcriptome Analysis Console (TAC, Affymetrix). Genes uploaded into Ingenuity Pathway Analysis (IPA; Qiagen Bioinformatics; Redwood City, CA, United States) for pathway analysis were defined as significantly altered with a *p*-value ≤ 0.05 and absolute fold change ≥ 1.5. Gene expression was validated by qPCR of 22 genes of interest. The validation and IPA analysis data was reported in Baker et al. (2016). Although all microarray data were previously uploaded, fold changes for a subset of genes not reported in the previous paper are discussed here for the first time.

### Whole Genome Bisulfite Sequencing

Bisulfite conversion of methylated cytosines of DNA from zebrafish testes was performed using the EZ DNA Methylation Kit (Zymo, Irvine, CA, United States). The resulting bisulfite-converted ssDNA was converted to Illumina libraries with the TruSeq DNA Methylation Kit (Illumina, San Diego, CA, United States). DNA libraries were sequenced with 100 bp paired-end reads on an Illumina HiSeq 2500 run in high output mode. One of the TCDD exposed samples did not pass our quality control cutoffs post amplification and was removed from the study. Reads from remaining four control and three TCDD-treated fish were aligned (bismark 0.18.1; bowtie2 2.2.3) to the zebrafish genome (dR10) and differentially methylated sites were determined (methylKit 1.6.3) between conditions (Supplementary Table S2) (Langmead et al., 2009; Krueger and Andrews, 2011; Akalin et al., 2012). Methylated sites (percent methylation change > 5%; *p*-value ≤ 0.05) were annotated to the nearest gene. 5% methylation change was used as a cutoff after referencing previous studies that used a 5% cutoff for significant methylation change or considered changes less than 5% significant (Yang et al., 2006; Byun et al., 2013; Dayeh et al., 2014). Differentially expressed genes from the microarray data were overlapped with annotations for differentially methylated sites. Individual replicates from each condition were kept separate and not pooled.

### Ingenuity Pathway Analysis

Genes associated with differential methylation (methylation change > 5%, *p*-value ≤ 0.05) were converted to homologous human genes and uploaded into IPA software and analyzed using RefSeq ID as the identifier. Fifty-three molecules were available for pathway analysis of enriched disease and biologic functions (Supplementary Table S4).

## Results

### DNA Methylation

#### CpG Sites

All DNA methylation data can be found in Supplementary Table S3. WGBS of control and TCDD-treated zebrafish revealed that 12.38% of total cytosines were methylated in control samples and 12.57% were methylated in TCDD exposed samples. In a CpG context, 86.55% of cytosines were methylated in control samples and 87.57% of cytosines were methylated in TCDD exposed samples. These changes in global methylation are not statistically significant with *p*-values of 0.48 for total cytosines and 0.54 for cytosines in a CpG context. There were, however, significant alterations in DNA methylation in specific genomic locations between the control and exposed groups.

**FIGURE 1:**
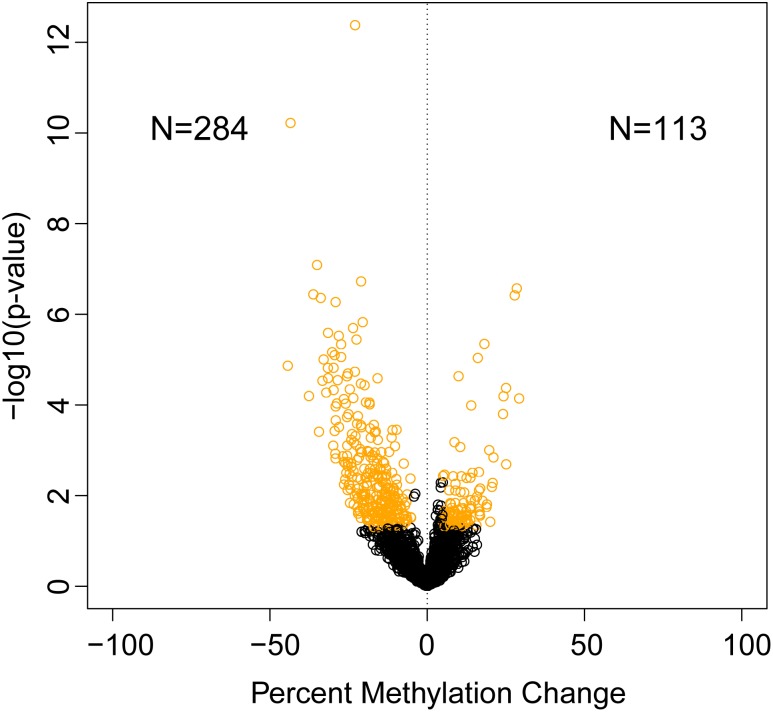
Identification of significantly differentially methylated sites between dioxin treated and untreated zebrafish gonads. Volcano plot depicting percent methylation change and *p*-value of methylated sites (circles). Significantly changed CpG sites (percent methylation change 5%; *p*-value ≤ 0.05) are in yellow.

**FIGURE 2:**
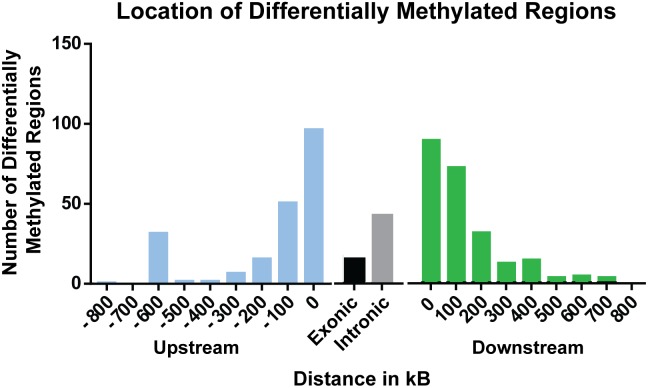
Distribution of significant (percent methylation change ≥5%; *p*-value ≤ 0.05) differentially methylated sites relative to nearest gene.

A total of 397 sites were significantly differentially methylated between control and exposed fish. Of these, 284 were hypomethylated and 113 were hypermethylated (Figure 1). Of these differentially methylated sites, 89 sites were found <100 kb downstream of genes, followed by 97 sites <100 kb upstream of genes. Within genes, more differentially methylated sites were found within introns (42) rather than within exons (15) (Figure 2).

Differentially methylated genes of interest related to reproduction, RNA regulation, and epigenetic processes are listed in Table 1. Of the genes related to reproduction, *bambia*, *chrna4*, *hug*, and *fmr1* are all involved in spermatogenesis, which is consistent with our previous studies that observed defects in spermatogenesis in response to TCDD exposure. *Fmr1* is also involved in RNA regulation as are *dhx36*, *klf15*, and *pym1*, while *eya3*, *wdr5*, and *zgc:162967* are involved in histone modification.

**Table 1:** Genes with significant changes in DNA methylation (*p*-value <0.05, methylation change >5%) that are involved in reproduction, RNA regulation, or epigenetic regulation.

Gene symbol	Full gene name	Role	Methylated sites	DMRs
**Reproduction**				
*bambia*	BMP and activin membrane-bound inhibitor a	Conserved in trout/mice spermatogenesis	2	
*chrna4b*	Cholinergic receptor, nicotinic, alpha 4b	Involved in signal transduction during spermatogenesis and sperm motility	1	
*clcn2a^∧^*	Chloride channel, voltage-sensitive 2a	KO mice develop sterility	1	2
*dnaja2*	DnaJ heat shock protein family (Hsp40) member A2	Involved in spermiogenesis and androgen signaling		2
*dync2li1*	Dynein, cytoplasmic 2, light intermediate chain 1	Associated with testis abnormalities	2	
*elavl1*	ELAV like RNA binding protein 1	Deletion can lead to male sterility; critical to spermatogenesis	4	
*fmr1^∧^*	Fragile X mental retardation 1	Regulates DNA damage response during spermatogenesis	2	6
*lztfl1*	Leucine zipper transcription factor-like 1	Non-sense mutations can lead to hypogenitalism, micropenis, atrophic testes	2	
*mc4r*	Melanocortin 4 receptor	Involved in hyperinsulinemia, LH release, ovulation; KO leads to infertility	1	
*ndufc2^∧^*	NADH: Ubiquinone oxidoreductase subunit C2	Impacts fertility through involvement in the electron transport chain	1	2
*nr1h3*	Nuclear receptor subfamily 1, group H, member 3	Transcription factor involved in the regulation of testis physiology	3	
*stoml2^∧^*	Stomatin like 2	Impacts fertility through mitochondrial biogenesis	4	7
**RNA regulation**				
*dhx36^∧^*	DEAH-box helicase 36	mRNA degradation, deadenylation; binds/resolves RNA/DNA quadruplex	1	2
*fmr1^∧^*	Fragile X mental retardation 1	Involved in RNA nuclear export	2	6
*klf15*	Kruppel like factor 15	Transcriptional activator activity, RNA polymerase II proximal promoter	1	
*pym1^∧^*	PYM homolog 1	Involved in mRNA export, non-sense-mediated mRNA decay, and translation	3	3
**Epigenetic regulation**				
*eya3*	EYA transcriptional coactivator and phosphatase 3	Dephosphorylates H2AX	1	
*riox1*	Ribosomal oxygenase 1	Histone demethylase	2	
*wdr5*	WD repeat domain 5	Histone methyltransferase; interacts in complex with setd1a/1b	1	


*^∧^indicates genes associated with both individual methylation sites and one or more DMRs.*

#### Differentially Methylated Regions

**FIGURE 3:**
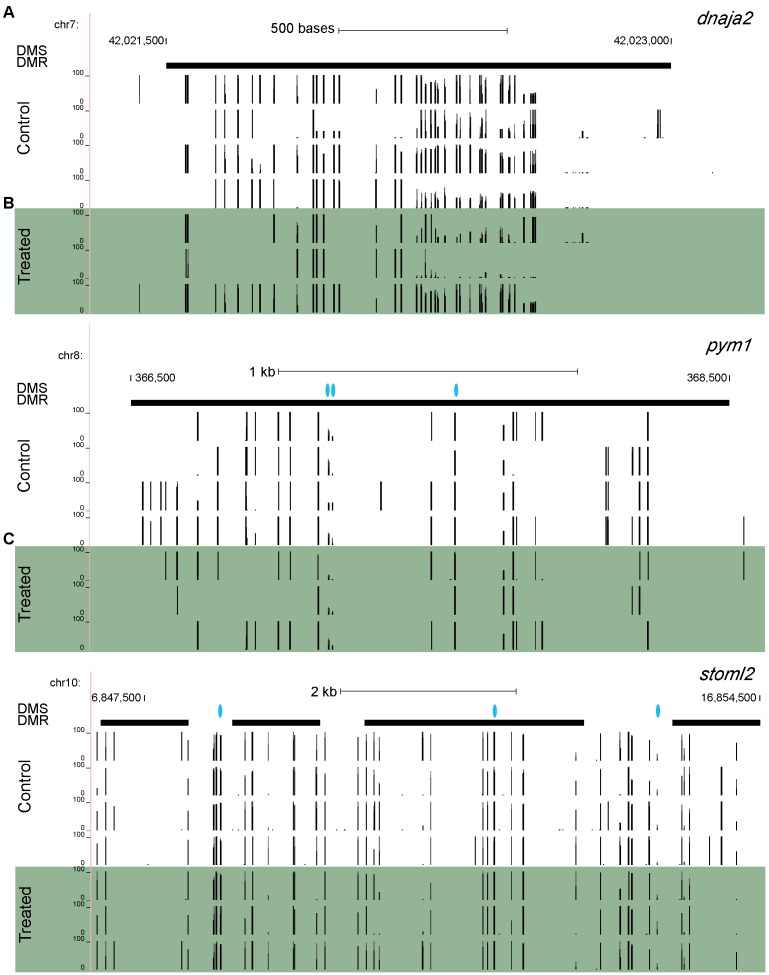
Maps of individual methylated sites linked to three genes: **(A)** *dnaja2*, **(B)** *pym1*, and **(C)** *stoml2*. Vertical black bars indicate individual DNA methylation sites and height of the bars represents methylation value. Blue dots represent differentially methylated CpG sites, and bolded horizontal black bars represent differentially methylated regions. Each row represents a different sample.

In addition to individual CpG sites, we also discovered differentially methylated regions (DMRs). At a window size of 1,000 bp and a step increment of 500 bp, there were a total of 148 statistically significant (*p*-value < 0.05) DMRs linked to 64 different genes (several DMRs were linked to the same gene). Maps of individual methylated sites linked to three genes of interest are shown in Figure 3. 41 CpG-linked genes were also linked to at least one DMR, and 23 DMR-linked genes were found that were not linked to any individual CpG sites; for example, the reproductive gene *dnaja2* (Figure 3A). Notably, several reproductive genes are linked to both individual CpG sites as well as DMRs: *fmr1*, *ndufc2*, and *stoml2* (Figure 3C). *Pym1* (Figure 3B) and *dhx36*, which are both involved in RNA regulatory mechanisms, were also linked to both CpG sites and DMRs. Finally, *crestin*, which is both differentially expressed and linked to a CpG site, is also linked to a DMR.

### Pathway Analysis

We performed IPA on genes linked to differentially methylated sites in TCDD exposed zebrafish testes and found 405 total associated diseases and annotation pathways. Reproductive system disease (*p*-values: 4.79E-02 – 1.46E-03), as well as reproductive development and function (*p*-values: 4.93E-02 – 1.46E-03) were reported under top diseases and biological functions. Two of the top networks affected included gene expression (Gene Expression, Embryonic Development, Nervous System Development and Function, Hypersensitivity) and cell cycle (Cell Cycle, Auditory Disease, Cellular Development). Table 2 shows the diseases or functions from IPA analyses that are involved in reproduction, gene expression regulation, or cellular morphology and development.

**Table 2:** Diseases or functions from IPA analysis of genes with significant changes in DNA methylation between control and TCDD-exposed groups that are involved in reproduction, gene expression regulation, or cellular morphology and development.

Diseases or functions annotation	*p*-value	Number of genes
**Reproduction**		
Abnormal morphology of enlarged testis	2.61E-02	1
Abnormal morphology of internal genitalia	2.67E-02	4
Cell death of male germ cells	4.23E-02	2
Delayed puberty	9.53E-03	1
Elongation of spermatids	1.43E-02	1
Fertility	2.82E-02	4
Proliferation of spermatogonia	4.01E-02	1
**Gene expression regulation**		
Dissociation of ribosome	2.40E-03	1
Expression of RNA	1.13E-02	15
Initiation of translation of mRNA	6.69E-03	2
Localization of mRNA	1.91E-02	1
Stabilization of mRNA	8.30E-03	2
Transcription	3.45E-02	13
Translation of mRNA	1.42E-02	3
**Cell morphology and development**		
Depolymerization of filaments	1.74E-02	2
Destabilization of microtubules	4.24E-02	1
Disappearance of focal adhesions	4.80E-03	1
Formation of cytoplasmic matrix	7.19E-03	1
Morphology of cellular protrusions	1.40E-02	4
Remodeling of actin	2.61E-02	1
Size of cells	7.94E-03	6


### Differentially Expressed Genes Involved in Epigenetic Regulation

Our microarray analysis identified five differentially methylated genes involved in epigenetic regulation (Table 3): *wdr5* (fold change: -1.26, *p*-value: 0.029), *setd1bb* (fold change: -1.31, *p*-value: 0.049), *dnmt5* (fold change: -1.49, *p*-value: 0.04), *dnmt3* (fold change: -1.44, *p*-value: 0.03), and *dnmt3b* (fold change: -1.32, *p*-value: 0.02). *Wdr5* and *setd1bb* are histone methyltransferases and *dnmt5*, *dnmt3*, and *dnmt3b* are DNA methyltransferases. *Wdr5* is likely involved in positioning lysine 4 of histone H3 for methylation and *setd1bb* trimethylates this site. These DNA methyltransferases are involved in *de novo* DNA methylation which occurs during development, as opposed to maintenance methylation.

**Table 3:** Genes from microarray with significant differential expression with roles in epigenetic regulation.

Gene expression			
	
Gene	Fold change	*p*-value	Role
*wdr5*	-1.26	2.91E-02	H3K4 histone methylation regulator
*setd1bb*	-1.31	4.95E-02	H3K4 histone methyltransferase
*dmnt5*	-1.49	4.46E-02	*De novo* DNA methyltransferases
*dmnt3*	-1.44	3.40E-02	
*dmnt3b*	-1.32	2.29E-02	


### Relationship Between DNA Methylation and Gene Expression

Genes with proximally modified DNA methylation sites and differential expression between TCDD-exposed and control testes are displayed in Table 4. The differentially methylated sites near *riox1*, *crestin*, *wdr5*, and *sox21b* are upstream of their respective genes while the differentially methylated sites near *flrt2*, *rab11bb*, and *gabra1* are located downstream of their respective genes.

**Table 4:** Genes with significant differential expression and proximal changes in DNA methylation status.

	Methylation		Gene expression	
		
Gene	Percent change	*p*-value	Log fold change	*p*-value
*crestin^∗^*^,^ *^∧^*	-31.6	1.52E-05	-1.34	4.79E-02
*flrt2^∗^*	6.83	2.38E-02	1.20	4.26E-02
	8.38	2.78E-02		
	8.95	7.67E-03		
*rab11bb^∗^*	-10.1	3.81E-02	1.34	1.85E-03
	12.2	3.92E-02		
*riox1^∗^*	-18.6	1.07E-03	-1.28	3.57E-02
	10.7	2.76E-02		
*wdr5^∗^*	-25.7	3.14E-03	-1.26	2.91E-02
	-20.0	1.24E-02		
	-19.1	3.07E-02		
	8.80	2.25E-02		
	11.6	1.45E-02		
	12.5	3.35E-02		
*gabra1^∧^*	5.20	4.65E-02	-1.17	4.44E-02
*sox21b^∧^*	7.30	2.49E-04	1.14	3.74E-02
	8.97	5.51E-04


*Each line under methylation refers to a separate CpG site or DMR. ^∗^ indicates the gene is linked to a CpG site, and ^∧^ indicates that the gene is linked to a DMR.*

*Flrt2* and *crestin*, involved in embryonic development (Luo et al., 2001; Müller et al., 2011), and the transcription factor *sox21b*, involved in neuronal differentiation (Sandberg et al., 2005), have methylation changes directly related to gene expression. *Flrt2* has increased expression and hyper-methylation at three CpG sites; likewise, *sox21b* is upregulated and hyper-methylated at two associated DMRs. *Crestin* expression, however, is reduced, with hypo-methylation of both the neighboring CpG site and associated DMR. *Gabra1*, a GABA receptor subunit (Bhat et al., 2018) has decreased expression while the associated DMR is hyper-methylated, demonstrating a relationship between methylation and gene expression. *Riox1* and *wdr5*, respectively, a histone demethylase and histone methyltransferase (Aravind et al., 2011; Tao et al., 2013), exhibit decreased expression while the endosomal GTPase *rab11bb* (Clark et al., 2011) has increased expression, but all three are surrounded by a mixture of hyper and hypo-methylated sites (*riox* and *wdr5*) and DMRs (*rab11bb*).

## Discussion

Whole genome methylation analysis revealed both DMR- and CpG-specific changes in the DNA methylation profile of adult zebrafish developmentally exposed to sublethal levels of TCDD. Previously, we found that this exposure paradigm induced transgenerational adult infertility mediated through the male germ line (Baker et al., 2014a,b). As the exposure occurred during reproductive development, the latency to effect (3–10 months) and transmission of these outcomes across generations strongly implicate the presence of persistent epimutations in reproductive pathways as a mechanism for the infertility phenotype. Our promising findings of TCDD-induced differential methylation in reproductive disease, gene expression, and cell morphology and development pathways correspond with our phenotypic and transcriptomic outcomes and are not unexpected, considering recent findings that disruption of DNA methylation during gonad development can lead to changes in reproductive and epigenetic genes, such as increases in *dnmt1* in adult zebrafish (Ribas et al., 2017).

About two-thirds of DMR-associated genes overlap with individual CpG-associated genes, including genes involved in mitochondrial function. For example, *stoml2*, associated with a mix of hypo- and hyper-methylated sites, regulates mitochondrial biogenesis (Christie et al., 2011) along with *ndufc2*, a member of the electron transport chain (Stroud et al., 2016) associated with hypo-methylated regions. Dysregulation in mitochondrial pathways can impact multiple physiological processes, including reproductive health and fertility; thus, epimutations in these pathways have potential to impair fertility through several distinct mechanisms. Spermatogenesis is a closely controlled process with high energy demands, thus any shifts or deficits in energy balance due to altered quantity and output of gonadal mitochondria may arrest sperm cell maturation (Nakada et al., 2006). Additionally, impairment of *ndufc2*, as demonstrated in a rodent knock-out model, can induce mitochondrial damage and oxidative stress, which is known to damage the sperm membrane and induce infertility (Tremellen, 2008; Ramalho-Santos et al., 2009; Raffa et al., 2017). Another gene implicated in oxidative stress response, *dnaja2*, is an *hsp70* co-chaperone associated solely with hypo-methylated DMRs. *Dnaja2* plays a role in maintaining protein stability and regulating apoptosis; knockout of a closely related DnaJ homolog resulted in disrupted Sertoli-germ cell adherens junctions and severely impaired spermatogenesis, suggesting an important role in sperm development and androgen signaling (Terada et al., 2005; Meccariello et al., 2014).

Analysis of differentially methylated individual CpGs uncovered linked genes associated with several pathways dysregulated in infertility, including spermatogenesis and glucose/lipid metabolism. *Bambia*, associated with a mix of hypo- and hyper-methylated sites, and *chrna4b*, associated with a single hypo-methylated site, are implicated in the initiation and progression of spermatogenesis in germ cells and Leydig cells, respectively, suggesting that epimutations in these genes may contribute to the infertility phenotype (Loveland et al., 2003; Hamra et al., 2004; Bray et al., 2005; Ge et al., 2005; Rolland et al., 2009). Glucose and lipid metabolism, two components of energy metabolism, cooperate to regulate sex hormone production and initiation of gametogenesis (Fontana and Della Torre, 2016). CpG methylation changes linked to several metabolism genes, namely *klf15*, a transcription factor involved in glucose homeostasis, lipid accumulation, and testosterone production (Gray et al., 2002; Du et al., 2009), and *nr1h3*, which controls cholesterol homeostasis and steroidogenic gene expression (Zhao and Dahlman-Wright, 2010), suggest dysregulation of the tightly regulated feedback loop between the glucose and lipid metabolism pathways and the reproductive system. *Klf15* is linked to a hyper-methylated CpG site, whereas *nr1h3* is linked to a mixture of hyper- and hypo-methylated sites. An imbalance in these pathways can result in outcomes such as impaired gonadal somatic (Leydig and Sertoli) cell function, decreased sperm quality, and subsequent infertility (Alves et al., 2013; Ding et al., 2015; Parhofer, 2015), aligning with our phenotypic and transcriptomic results of decreased fertilization, a shift in germ cell ratio toward less mature cells, and altered expression of genes linked to spermatogenesis, testicular development, lipid metabolism, and steroidogenesis pathways (Baker et al., 2016).

In addition to reproductive genes, we observed differential methylation of a subset of histone methyltransferases, histone demethylases, miRNAs, and RNA modifying genes. Although histone methylation interacts both independently and reciprocally with DNA methylation in controlling gene expression and chromatin state (Rose and Klose, 2014), few studies have assessed the impact of TCDD exposure on histone methylation. Of particular interest is *wdr5*, a core component of several histone methyltransferase complexes, including the SET1 complex, which primarily modulates methylation of the classic gene activation mark H3K4 (Barski et al., 2007; Aravind et al., 2011). Along with a crucial role in global gene activation, *wdr5* is involved in programming embryonic stem cell differentiation and self-renewal (Ang et al., 2011), as well as epigenetically controlling gluconeogenic pathways (Ravnskjaer et al., 2013), that are implicated in fertility and reproduction. *Wdr5* and *riox1*, a histone demethylase, are both differentially methylated (mixture of hypo- and hyper-methylated CpG sites) and downregulated, suggesting that their methylation state and expression profile are directly linked, and that decreases in histone methyltransferase and demethylase function are contributing to the fertility-linked epimutations present in the genome.

Transcriptomic data also confirms TCDD-induced downregulation of several other critical histone and DNA methyltransferase genes, including the histone methyltransferase *setd1bb*, involved in H3K4 methylation alongside *wdr5*, and the *de novo* DNA methyltransferases *dnmt3*, *dnmt3b*, and *dnmt5*, involved in establishment of DNA methylation patterns during gametogenesis (Campos et al., 2012). As histone and DNA methyltransferases mediate the methylome, toxicant-induced changes in their expression could account for the changes in methylation pattern. Decreases in DNA and histone methyltransferase activity would generally be expected to result in global and/or gene-specific hypomethylation and subsequent increases in gene expression. However, we found no global hypomethylation and very limited site-specific hypomethylation/corresponding upregulation of gene expression. These outcomes align with Aluru et al. (2015), which reported decreases in several dnmt3 genes (*dnmt3a1*, *3b1*, and *3b4*) after developmental exposure to TCDD in zebrafish, and both hyper- and hypo-methylation in the promoter region of AhR target genes that overall did not correlate with gene expression. Somm et al. (2013) also reported limited relationships between gene expression and methylation of imprinted genes in the sperm of male mice gestationally exposed to TCDD. Conversely, both Papoutsis et al. (2015) and Sekaran and Jagadeesan (2015) found that gestational exposure of rodents to TCDD and DEHP, respectively, resulted in gene-specific hyper-methylation and corresponding decreases in gene expression, with increased Dnmt levels reported by the latter. These outcomes support a multifaceted interpretation of the methylation-gene expression relationship, suggesting not only a variable, gene-dependent response, but also the possible involvement of multiple epigenetic processes in mediating transcriptomic response to toxicant insult. Results showing that the degree of H3K4 methylation can regulate *de novo* DNA methyltransferase activity (Rose and Klose, 2014) strengthen the link between DNA and histone methylation as potential mechanisms of epimutation with respect to our methylomic and transcriptomic findings. However, as the location and extent of histone methylation in TCDD-exposed fish remains to be studied, inferring further directional relationships from the complex interplay between DNA methylation, histone methylation, and gene expression remains a challenge. Several miRNAs, including *mir2185-1*, are repressive epigenetic factors that interact with histone modification and DNA methylation that were also differentially methylated by TCDD exposure, and may thus contribute to modifications of the transcriptome (Silahtaroglu and Stenvang, 2010; Osella et al., 2014). *Mir2185-1* is associated with several hyper-methylated DMRs and a mix of hyper- and hypo-methylated individual CpGs. Taken together, TCDD-induced differential methylation of epigenetic genes has potential to alter both genome-wide and pathway-specific patterns of methylation through a variety of inter-related mechanisms, leading to transcriptomic changes and infertility.

Several differentially methylated genes were implicated in the regulation of gene expression. *Fmr1*, *pym1*, and *dhx36* are associated with both differentially methylated individual CpG sites and DMRs, whereas *elavl1* is specifically associated with individual CpG sites. Although not epigenetic factors by definition, these RNA-modifying genes regulate the transcriptome and proteome; therefore, altered methylation and potentially altered expression of these genes can have broad consequences for overall gene transcription and translation (Bächner et al., 1993; Diem et al., 2007; Chi et al., 2011; Ferder et al., 2013; Booy et al., 2014). In some cases, the regulatory function of these genes directly impacts reproductive outcomes; for example, post-transcriptional activity of *elavl1* is essential for both meiotic division and spermatid differentiation, thus leading to extensive gonadal cell death and subsequent azoospermia in a mouse knockout model (Chi et al., 2011). Likewise, *fmr1* interacts with chromatin to regulate DNA damage during meiosis, resulting in macroorchidism, impaired spermatogenesis, and maturation arrest when downregulated (Slegtenhorst-Eegdeman et al., 1998; Tian et al., 2013; Alpatov et al., 2014). However, the majority of these genes (excepting *dhx36*) are linked to a mix of hyper- and hypo-methylated sites, posing a challenge in the interpretation of downstream effects. Overall, differential methylation of these genes corresponds with our findings of dysregulation of cell morphology and development pathways, with potential for broad defects in gonadal structure and function leading to infertility outcomes in adulthood (Baker et al., 2016).

No global shift in methylation was detected; however, a substantial number of site-specific changes were present, highlighting the advantage of WGBS as a genome-wide, high-specificity method. EDC exposure has both global and gene-specific effects on the methylome (Anway et al., 2005; Bollati et al., 2007; Dolinoy et al., 2007; Pilsner et al., 2007; Onishchenko et al., 2008), but outcomes vary widely depending on targeted pathways and critical windows of exposure. TCDD-mediated AhR recruitment of methyltransferase enzymes to specific loci (Papoutsis et al., 2015; Alavian-Ghavanini and Rüegg, 2018) may underlie our site-specific findings.

About 71% of differentially methylated genes were hypomethylated in adulthood as a result of developmental TCDD exposure; this corresponds with reports of AhR and nuclear receptor-driven site-specific CpG demethylation (Métivier et al., 2008; Amenya et al., 2016) and aligns with our findings of transcriptomic downregulation of DNA and histone methyltransferase genes. Our work demonstrates that 69% of differentially methylated sites were located within 100 kb of linked genes, with about 9% located within 10 kb. Although promoter methylation is commonly associated with differential gene expression, McGaughey et al. (2014) found the pattern of methylation 10 kb upstream or downstream of a gene to be more predictive of gene expression than promoter methylation status, suggesting a broadened, more complex view of the interplay between methylome and transcriptome.

Despite the overall similarities in affected transcriptomic and methylomic functional pathways in TCDD-exposed fish, seven genes are both differentially expressed and differentially methylated. One gene involved in neural crest development, *crestin*, is linked to both individual CpGs and DMRs, while the GABA receptor subunit *gabra1* and neuronal transcription factor *sox21b* are associated specifically with DMRs. The remaining four genes are associated only with individual CpG sites: histone-modifying genes *wdr5* and *riox1* as mentioned above, along with the endosomal GTPase *rab11bb* and *flrt2*, involved in embryonic development. This is not wholly unexpected considering prior literature, as other studies exposing zebrafish embryos to TCDD resulted in limited genome-wide alterations in methylation and did not demonstrate an inverse relationship between gene expression and promoter methylation (Olsvik et al., 2014; Aluru et al., 2015). It is possible that other epigenetic or regulatory effects are controlling this interaction. This disparity also suggests a tightly regulated temporal aspect to dysregulation of reproductive pathways: exposure to TCDD during critical periods alters the methylome, which shifts the careful interplay of rapid gene activation and repression during early life (Nagy and Turecki, 2012; Piferrer, 2013). Although such transcriptomic effects may not linger into adulthood, there may be lifelong consequences of disrupting development-specific gene programming, including changes to gonadal histology that present in adult fish or shifts in the sensitivity and response of the hypothalamic–pituitary–gonadal axis. Another factor that may contribute to the minimal overlap between differentially expressed and differentially methylated genes is the assessment of whole testes. As testicular tissue is a heterogeneous mixture of germ cells at various stages of maturity with supporting Leydig and Sertoli cells, taking sperm samples or sorting testicular cell populations prior to analysis may help prevent any potential masking of transcriptomic and methylomic effects. Future directions include single cell analysis and further investigations of these potential relationships by qPCR and pyrosequencing.

As exposure to TCDD occurred during juvenile development, detection of differential methylation in reproductive pathways well into adulthood, taken together with the transgenerational infertility phenotype, suggests persistent epimutations that may be inherited in the F_1_ or F_2_ generations. DNA methylation, although a potential mechanism for the phenotypic and transcriptomic effects previously demonstrated, is only one of several epigenetic factors, which also include histone modification and microRNA, that cooperate to regulate the transcriptome (Sun et al., 2013; Alavian-Ghavanini and Rüegg, 2018). Differential methylation and transcription of several DNA methylation, histone methylation, and miRNA genes suggests that multiple epigenetic mechanisms interact with each other and with the transcriptome to mediate toxicant-induced infertility in zebrafish, establishing the need for future studies to investigate these mechanisms and determine promising methods of clinical intervention for adult-onset and transgenerational reproductive disease.

## Author Contributions

CA and DM wrote the manuscript, generated the tables, and performed the IPA analysis. TB edited the manuscript, performed the experimental exposures, dissections, tissue collection, and analyzed the microarray data. KG analyzed all WGBS data and generated the corresponding figures.

## Conflict of Interest Statement

The authors declare that the research was conducted in the absence of any commercial or financial relationships that could be construed as a potential conflict of interest.

## Supplementary Material

Table S1 Concentrations, A260/A230, and A260/A280 of DNA after tissue isolation, bisulfite conversion, and amplification.

Table S2 Mapping efficiency, total C analyzed, percent C methylated, total CpG methylated and percent C methylated in CpG context for control and TCDD-treated DNA.

Table S3 Data for each differentially methylated site including genomic location, *p*-value, *q*-value, methylation difference, distance to nearest gene, and location and annotation of nearest gene.

Table S4 Diseases and biological functions results from Ingenuity Pathway Analysis (IPA) of genes with significant changes in DNA methylation between control and TCDD-exposed groups.
